# Risk factors of a viral nervous necrosis disease in grouper (*Epinephelus* spp.) cultured in Bintan district, Indonesia

**DOI:** 10.14202/vetworld.2018.1558-1563

**Published:** 2018-11-09

**Authors:** Eka Juniar, Kurniasih Kurniasih, Bambang Sumiarto

**Affiliations:** 1 Postgraduate Student of Veterinary Science, Faculty of Veterinary Medicine, Gadjah Mada University, Yogyakarta 55281, Indonesia; 2 Department of Pathology, Faculty of Veterinary Medicine, Gadjah Mada University, Yogyakarta 55281, Indonesia; 3 Department of Veterinary Public Health, Faculty of Veterinary Medicine, Gadjah Mada University, Yogyakarta 55281, Indonesia

**Keywords:** Grouper, multivariate analysis, risk factors, viral nervous necrosis

## Abstract

**Aim:**

The aim of the study was to determine the prevalence and risk factors of viral nervous necrosis (VNN) disease in tiger grouper cultured in the floating net cage in Bintan district, Indonesia.

**Materials and Methods:**

Sampling used multiple stages with ponds as interest units. The selection conducted by systematic random sampling from the entire net cage in Bintan district. The fish samples were selected based on the appearance of clinical signs of infected fish. The risk factors investigated in this study included net cage, technical, and sample fish information, culture, water quality, and feed management. A total of 195 fishes pooled to the 39 net cages and tested using the nested polymerase chain reaction technique to determine the VNN status. The brain and eye processed for histopathology.

**Results:**

The prevalence rate of VNN on the net cage was 38% (15/39). The risk factors affecting VNN using bivariate analysis was cleaning the net (*χ*^2^=9.80; p=0.002), replacement of net cage (*χ*^2^=5.20; p=0.0226), and floating net cage technicians knowledge (*χ*^2^=4.13; p=0.042). The variables of positive risk factors affecting VNN by multivariate analysis were the juvenile source and the level of mid-weather changes (technician experience and dissolved oxygen [DO]). Seven variables associated to the VNN outbreak have detected. The positive multiplier factors were the source of juveniles, mid weather changes, technician experience, and the DO, while the negative factors were salinity, mixed feed, and the low weather changes. Histopathologically, the grouper fish showed the brain, eye, and muscle vacuolization and kidney necrosis.

**Conclusion:**

It proves that the Bintan waters contaminated by VNN had a prevalence rate of 38% from the total sample based on bivariate method, net cleaning, net replacement, and knowledge on the VNN outbreak. Natural infection of VNN in grouper leads to vacuolization of the brain, the eye, and muscles nearby the eye as well the kidney necrosis.

## Introduction

Riau Island is the largest area in Indonesia that produces the grouper, with a total of 1940 ton production [1]. The juveniles supplied by the fisher or its hatchery [2]. Bintan district is the base of the grouper producer in the Riau, and its productivity reaches about 46% (892 ton/1940 ton) in 2013 [3]. Viral nervous necrosis (VNN) also was known as viral encephalopathy retinopathy; fish viral encephalitis is a neuropathogenic disease caused by Betanodavirus infection from the Nodavirus family. VNN has a spherical form with a diameter of 25-30 nm and non-envelope virus. VNN has a significant economic impact on the ocean’s industry since 1980 and causes massive deaths of >39 fish species [4,5] including grouper.

The first report of the VNN was identified in 1990 on a parrot fish (larval and juvenile stadium) in Japan and barramundi fish in Australia. At June–July in the Nagasaki, a large number of the juvenile with 6-25 mm lengths died when the water temperature increases during the summer [6,7] which then spread widely to the Southeast Asia region, Mediterranean countries, the United Kingdom, North America, and also Australia [8]. In Indonesia itself, VNN infects the snapper fish hatchery in East Java, in 1997, which then spreading to Ambon territorial waters [3,9].

The spreading of VNN disease in the Bintan mediated by the water, food, and spawns from an outside district [10,11] and the inappropriate handling [12]. The infection of VNN is difficult to eradicate since the environmental condition also plays a major role in viral growth and spreading [13]. An appropriate condition to support the growth of the grouper is water temperature (24-31°C), the salinity (30-33 ppt), dissolved oxygen (DO) (>3.5 ppm), and the pH (7.8-8.0) [14]. Another factor such as culture management and food is also related to fish illnesses [15]. Culture management at the cage net consists of stocking density, supervision, and routine cage maintenance. The high stocking density could raise the diseases probability and triggers cannibalism among fishes [16].

The aim of the study was to determine the prevalence and risk factors of VNN disease in tiger grouper cultured in the floating net cage in Bintan district, Indonesia.

## Materials and Methods

### Ethical approval

Ethical approval with certificate no. 00034/04/LPPT/IV/2017 issued by LPPT Gadjah Mada University.

### Sample size

The sample collected in the hatchery unit by the double stages method and mapped by the assumption of the prevalence of 15%, the confidence level of 95%, and the maximum error of 0.05. The sample size estimated by the following formula (n=4 PQ/L^2^) [17].

Based on the formula, the sample (n) discovered about 204. Then, the sample size was recalculated using the design effect approach. The fish pond separated into 49 areas with a total of five fish from each. It collected by the systemic random method following the purposive samples based on its clinical signs.

### Sample analysis of fish

This study used a target organ including 195 grouper fish brain. The samples examined by the pooling method to become 39 pools. The organ was extracted with RNeasy Mini Kit (Qiagen) and amplified with Qiagen nested polymerase chain reaction (PCR)using the suggested specific primer by Gomez *et al*. [18].

The eye and kidney fixed with 10% neutral buffer formalin, dehydrated using the tissue processor, blocked with paraffin, sectioned with a microtome into 5-7 µm of thickness, and stained with hematoxylin and eosin. The histopathological slides were examined using the light microscope.

### Statistical analysis

The prevalence of VNN analyzed using the formula below:


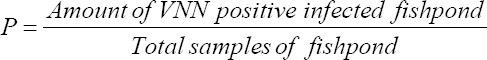


The detection of VNN conducted by analyzing the sample by describing data and finding the pattern of every variable. The relationship between two different variables, three, or more variables was analyzed by the bivariate and multivariate method. Univariate analysis conducted and interpreted on every variable. The bivariate analysis was conducted by Chi-square (χ^2^) test using the 2×2 table to determine the association between the cause and effect. The multivariate analysis was conducted to determine the risk factors as an independent variable that affects the dependent variable. All the findings analyzed by the statistical software version 8(Forbes Analytic Inc., USA) using the logistic regression method.

## Results

**Figure-1:**
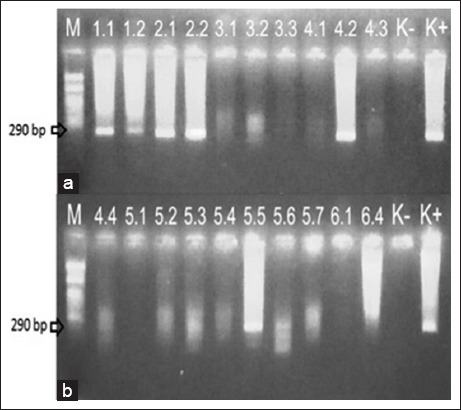
(a and b) The results of nested polymerase chain reaction (M=Marker; 1.1-6.4=The sample codes; K-=Negative control; K+=Positive control).

**Figure-2:**
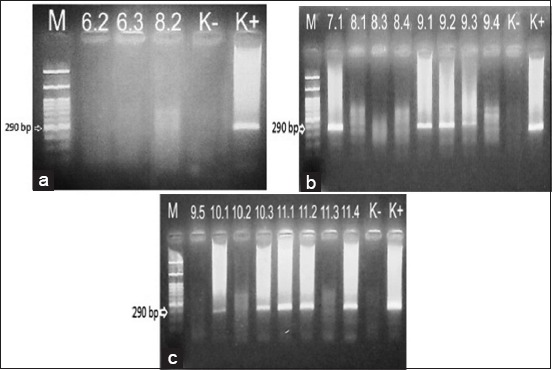
(a-c) The results of nested polymerase chain reaction (M=Marker; 6.2, 6.3, 8.2, and 7.1-11.4=Sample codes, K=Negative control, K+=Positive control).

Samples were taken from fish with erratic swimming behavior such as whirling and staying on the base of the pond. It supported by the nested PCR diagnostic test that shows 15 ponds are positive for VNN (Figures-1 and 2).

**Figure-3:**
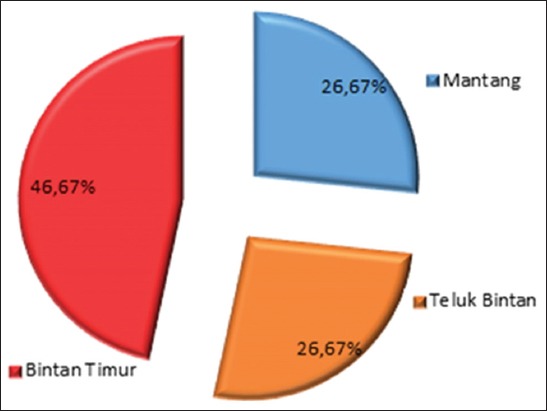
Proportion of prevalence on the subdistrict.

The prevalence of VNN on grouper in Bintan district was 38% (15/39). The highest is in Bintan subdistrict (46.67%) followed by East subdistrict and Mantang subdistrict (26.67%). The proportion caused by the number of samples (reach six groups) and the geographical location of Bintan subdistrict as a cultivation center in the Bintan district (Figure-3).

### Univariate variable description

The sampling method based on the cluster of the floating net cage, it obtained more or less than the target. The sampling distribution location was Mantang subdistrict seven ponds (17.95%), Bintan subdistrict 24 ponds (61.55%), and East Bintan subdistrict eight ponds (20.52%). Mostly, the selected ponds used as sampling sites were a private unit of 64.1% (25/39), while the others 35.9% (14/39) were government property cage units. The total of 56.4% (22/39) sampling sites indicates the prevalence of disease.

Mostly, the academic level of the technicians in the floating net cage was lower or equal to the junior high school. 20 out of 39 technicians (51.30%) were elementary school graduate, and the rest (48.70%) were secondary school graduate. Another finding shows that 56.4% (22/39) of the total technician has a working experience of fewer than 10 years, while 43.6% (17/39) has >10 years. Surprisingly, a total of 53.8% (21/39) of the technician in Bintan have a good knowledge of the VNN disease. It obtained by the counseling 81.9% (17/21) and the rest by their own experiences 19.2% (4/21).

The private cage unit was the primary sources of the juveniles 51.3% (20/39), while the government property cage unit only 48.7% (19/39). The juvenile producers that already had a guarantee certificate for the VNN disease free are about 71.8% (28/39); however, 28.2% (11/39) had not. The age of fish taken as the sample was ≤60 days 71.8% (28/39) and >60 days was 28.2% (11/39).

The emergence of pests and predators (PP) occurred in 97.4% (38/39) of floating net cage units, and 2.6% (1/39) did not appear. PP were birds and barnacles. The process of cleaning and replacing nets was also to eliminate competitor pests. Net cleaning was generally done once a week 74.4% (29/39), but there was also a net cleaning once every 2 weeks 25.6% (10/39). The net replacement has done once a month with percentage of 92.3% (36/39), while those who changed the net every 2 months 7.7% (3/39). Feeding frequency is 1-2 times a day showed 25.6% (10/39) and feeding 3-4 times daily as much as 74.4% (29/39). Technicians used natural and artificial feed during maintenance of 56.4% (22/39), artificial feed only of 41% (16/39), and only natural feed of 2.6% (1/39).

Daily water quality checking was performed in 7 out of 39 (17.9%) floating net cages, while the water quality of the rest of floating net cages (82.1%) are not checked daily. It is due to the unavailability of water quality measurement tools in every unit of the floating net cage. Water quality and the cage location were a prominent aspect of the aquaculture. The optimal water quality for the grouper expressed is shown in Table-1.

**Table-1:** Variable of waters quality management.

Variable	Information	Identification
pH	pH at the sampling site	6.74±2.05
DO	DO at the sampling site	8.25±3.59
Temperature	Temperature at the sampling site	30.00±0.42
Salinity	Salinity at the sampling site	26.49±8.49
Ammonium	Ammonium at the sampling site	0±0
Nitrite	Nitrite at the sampling site	0±0

### Bivariate variable description

The associations between net cleaning, replacement, and the technician’s knowledge variables against the VNN positive as the dependent variable were analyzed using the Chi-square (χ^2^) (Table-2).

**Table-2:** Associations between variable of risk factor with VNN positive.

Variable	Information	VNN		*χ*^2^	p-value

		+	−		
Net cleaning	Cleaning the net cage				
	1 week 1 time	7	22	9.80	0.002***
	2 weeks 1 time	8	2		
Net replacement	Replacement of the net				
	1 month 1 time	12	24	5.20	0.023**
	2 months 1 time	3	0		
Knowledge of VNN	Knowledge of VNN				
	Yes	5	16	4.13	0.042**
	No	10	8		

** Significant (p<0.05),

*** Very significant (p<0.01), VNN=Viral nervous necrosis

Factors that significantly influence (Table-2) were shown with values p=0.05, <0.05, and <0.01. Nets cleaning risk factors were a variable that relates very highly significant (*χ*^2^=9.80; p=0.002) with the prevalence of VNN in fish samples. The factors affect the VNN disease showed by p<0.05. Net cleaning becomes the highest risk factor variable to the prevalence of VNN in Bintan district (*χ*^2^=9.80; p=0.002), and it followed by the net replacement factors (*χ*^2^=5.20; p=0.0226) and technician’s knowledge of VNN (*χ*^2^=4.13; p=0.042). Those risk factors are affected by the presence of pests and predators (PP) in the sea. The technicians with good knowledge of VNN could more easily recognize the symptoms of VNN infection make it faster to take preventative management.

Based on questionnaires and field observations, the risk factors of the VNN disease in Bintan were classified into 27, such as floating net cage, technician, and fish information, maintenance, water quality, and feed management. Further, analysis was done by logistic regression test (Table-3).

**Table-3:** Logistic regression analysis of VNN disease model.

Predictor variables	Coefficient	SE	Coefficient/SE	p-value
Constant	12.7452	4.96754	2.57	0.0103
Origin	9.53477	3.77262	2.53	0.0115
Weather 1	−22.6712	8.31859	−2.73	0.0064
Weather 2	5.29581	2.94194	1.80	0.0718
DO	1.47644	0.61298	2.41	0.0160
Feed 2	−17.0412	6.28607	−2.71	0.0067
Technician experiences	3.51900	2.02221	1.74	0.0818
Salinity	−0.83096	0.28139	−2.95	0.0031

SE=Standard error, VNN=Viral nervous necrosis, DO=Dissolved oxygen

The model or multivariate analysis obtained from the logistic regression was VNN = 12.7452+9.53477 (origin)+5.29581 (weather 2)+3.51900 (technician experiences) +1.47644 (dissolved oxygen)–0.83096 (salinity)-17.0412 (feed 2)–22.6712 (weather 1).

The water salinity was the most influences variable on the VNN outbreak (−0.83096) in Bintan with p=0.0031 and odds ratios (OR) of 0.44 (OR=0.25-0.76). It shows that salinity reduces the VNN incidence by 0.44 times. Except for the salinity, weather change is the second influences variable affects the incidence of VNN with p=0.0064 and OR=−22.6712, while, the moderate weather changes associated to the VNN incidence (p=0.0718; OR=5.29581). The third factor was the grouper’s diet that has an impact on the VNN incidence (p=0.0067, OR=0.09-1.47). It proves that the artificial diet reduced the VNN incidence in the floating net cage 0.36 times smaller than the natural.

### Histopathology

**Figure-4:**
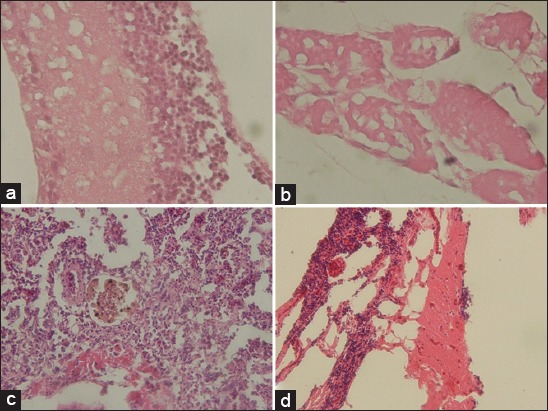
Histopathological changes of grouper organs with natural infection of viral nervous necrosis disease. (a) Vacuolation of the eye, (b) vacuolation of muscle near the eye, (c) necrosis of kidney from red snapper fish, and (d) vacuolation of the brain.

Samples of fish with a positive result by the molecular test were examined based on histopathology. Grouper fish showed the presence of vacuolization on the brain (Figure-4d), on the eye (Figure-4a), on the muscle near the eye (Figure-4b), and also necrosis of kidney (Figure-4c).

All the brain and eyes from fish, either stripped jack or Japanese jack mackerel, that infected by a stripped jack nervous necrosis virus (SJNNV) were positive by fluorescent antibody technique method [19]. The histopathological result of Indonesian grouper samples was similar to SJNNV infection in Japan.

## Discussion

The previous study showed that the VNN infection in grouper has several clinical signs such as erratic swimming behavior, hypersensitive to the stimulation, and loss of the appetite [20]. However, the VNN can inhibit by the maintenance of the water temperature (24-32°C), DO (>4 ppm), pH (6.5-9), salinity (15-35 ppt), ammonium (<0.1 ppm), and nitrite (<0.1 ppm) [21].

Waters in the tropical coastal with relatively high temperatures and small annual fluctuations submerged webs could be very rapidly passed by aquatic organisms so that if there was no regular cleaning could block the release of water through the net and caused low DO inside [22]. However, it was different from the water quality results in the study where the DO content was normal (8.25±3.59). The optimal dissolved DO for the growth of groupers was >5 ppm, whereas the temperature for maximum growth is 30°C [23].

Fish mortality caused by Betanodavirus varying depends on the fish health status and water condition. In the open water, the VNN transmission from one location to another was caused by tidal currents, boats coming from the infected area, and the carrier of the VNN such as the migratory wild fish [24]. Water was a prominent abiotic vector in the spreading of the VNN to nets, boots, and other cultivation equipment. Water mediates the VNN infection in the cultivated fish. During the infection period, VNN multiplies in the central nervous system tissues, and the infected fish serve as the horizontal infection to another [25]. The manifestation of the VNN infection in the brain and eyes such as vacuolization, necrosis, and inflammation reported by the previous study [8,19,26]. However, kidney and liver necrosis and inflammation found in the present study.

The environmental condition plays a prominent role in the fish defensive ability during the infection. The change of the environment leads to fish distress [27]. Based on this study, the VNN infection in the Bintan district affected by the several variables such as the source of the juvenile, weather change, technician experience, the water DO, and feed. Natural food could be one source of the transmission of VNN. Natural food often used in the grouper cultivation that could form of trash fish. According to Gomez *et al*. [11], trash fish and mollusks could be the source of the VNN in cultivated fish and caused high mortality until 90-100%.

## Conclusion

This study proves that Bintan water contaminated by the VNN with a total of the prevalence of 38%. There are three variables (net cleaning, net replacement, and technician knowledge) associated with the VNN based on the bivariate method. Based on the multivariate method, there are seven variables in two categories (positive and negative multiplier). The positive multiplier factor was the source of the juvenile, mid-weather change, technician experience, and DO, while the negative was salinity, mixed feed, and low weather changes. Natural infection of VNN on grouper in Bintan district causes the vacuolization of the brain, the eye, muscle near the eye, and necrosis and inflammation of the kidney.

## Authors’ Contributions

KK and BS participated equally in the study plan, and design. Sample collection from the floating net cage was done by EJ and KK. EJ and BS conducted the statistical analysis of data, EJ and KK reported of the results and collaborated in writing, revising, and improvising the article for publication. All authors read and approved the final manuscript.

## References

[1] Anonim (2013). Statistical Data of Annual Production of Indonesian Aquaculture.

[2] Anonim (1988). Budidaya Ikan Kerapu Lumpur (*Epinephelus tauvina*). Balai Informasi Pertanian.

[3] Novriadi R, Agustatik S, Bahri S, Sunantara D, Wijayanti DE (2014). Distribution of pathogen and environmental quality of marine fish farming in Riau Province. Depik J.

[4] Costa JZ, Thompson KD (2016). Understanding the interaction between betanodavirus and its host for the development of prophylactic measures for viral encephalopathy and retinopathy. Fish Shellfish Immunol.

[5] Doan QK, Vandeputte M, Chatain B, Morin T, Allal F (2017). Viral encephalopathy and retinopathy in aquaculture: A review. J. Fish Dis.

[6] Yoshikoshi K, Inoue K (1990). Viral nervous necrosis in hatchery-reared larvae and juveniles of Japanese parrotfish *Oplegnathus fasciatus*(Temminck and Schlegel). J. Fish Dis.

[7] Glazebrook JS, Heasman MP, dan DeBeer SW (1990). Picorna-like viral particles associated with mass mortalities in larval barramundi *Lates calcarifer* Bloch. J. Fish Dis.

[8] Shetty M, Maiti B, Santhosh KS, Venugopal MN, Karunasagar I (2012). Betanodavirus of marine and freshwater fish: Distribution, genomic organization, diagnosis and control measures. Indian J. Virol.

[9] Koesharyani I, Roza D, Mahardika K, Jhonny F, dan Yuasa K (2001). Penuntun Diagnosa Penyakit Ikan II. Balai Perikanan Laut Gondol.

[10] Anonim (2016). Viral Encephalopathy and Retinopathy 2.3.12. Manual of Diagnostic Tests for Aquatic Animals Disease.

[11] Gomez DK, Ichiro MK, Okinaka Y, Nakai T, dan Park SC (2010). Trash fish can be a source of betanodaviruses for cultured marine fish. Aquaculture.

[12] Setiadi CA (2016). Masuk Natuna, Virus VNN Mematikan. Tanjung Pinang Pos.

[13] Cameron A (1999). Survey Toolbox for Livestock Disease. A Practical Manual Dan Software Package for Active Surveillance in Developing Countries.

[14] Yoshimitsu T.H.E, Hiramatsu K (1986). Groupers Final Report Marine Culture Research and Development in Indonesia. ATA 192, JICA.

[15] Yanong R.P.E (2010). Viral Nervous Necrosis (Betanodavirus) Infections in Fish. IFAS Extension, IFAS Cooperative Extension Service, USA.

[16] Williams JB, McAllister PE, Smith G, Boston R (2002). Effect of fish density and number of infectious fish on the survival of rainbow trout fry *Oncorhynchus mykiss*(Walbaum), during epidemics of infectious pancreatic necrosis. J. Fish Dis.

[17] Martin SW, Meek AH, dan Willeberg P (1987). Veterinary Epidemiology-Principles and Methods.

[18] Gomez D, Baeck GW, Kim JC, Park SC (2008). Molecular detection of betanodavirus in wild marine fish populations in Korea. J. Vet. Diagn. Invest.

[19] Panzarin V, Cappelloza E, Mancin M, Milani A, Toffan A, Terregino C, Cattoli G (2014). *In vitro* study of the replication capacity of the RGNNV and the SJNNV betanodavirus genotypes and their natural reassortants in response to temperature. Vet. Res.

[20] OIE (2018). Viral Encephalopathy and Retinopathy.

[21] Ghufran M, Kordi H (2005). Budidaya Ikan Baronang.

[22] Chua TE (1979). Site Selection, Structural Design, Construction, Management and Production of Floating Cage Culture System in Malaysia. Proceedings of the International Workshop on Pen Cage Culture of Fish.

[23] De M, Ghaffar MA, Bakar Y, Das SK (2016). Effect of temperature and diet on growth and gastric emptying time of the hybrid *Epinephelus fuscoguttatus*♀×*E. lanceolatus*. Aquac. Rep.

[24] Nuñez-Ortiz N, Pascoli F, Picchietti S, Buonocore F, Marica T, Scapigliati G, Bernini C (2016). A formalin-inactivated immunogen against viral encephalopathy and retinopathy (VER) disease in European sea bass (*Dicentrarchus labrax*): Immunological and protection effects. Vet. Res.

[25] Nishioka T, Sugaya T, Kawato Y, Koh M, Nakai T (2016). Pathogenicity of striped jack nervous necrosis virus (SJNNV) isolated from asymptomatic wild Japanese jack mackerel *Trachurus japonicus*. J. Fish Pathol.

[26] Bovo G, Gustinelli A, Quaglio F, Gobbo F, Panzarin V, Fusaro A, Mutinelli F, Caffara M, Fioravanti ML (2011). Viral encephalopathy and retinopathy outbreaks in freshwater fish farmed in Italy. Dis. Aquat. Org.

[27] Afrianto E, Liviawaty E (1992). Pest Control and Fish Disease. Cetakan Pertama.

